# Dissimilarity Metric Based on Local Neighboring Information and Genetic Programming for Data Dissemination in Vehicular Ad Hoc Networks (VANETs)

**DOI:** 10.3390/s18072320

**Published:** 2018-07-17

**Authors:** Daniel Gutiérrez-Reina, Vishal Sharma, Ilsun You, Sergio Toral

**Affiliations:** 1 Engineering Department, Loyola Andalusia University, 14004 Seville, Spain; dgutierrez@uloyola.com; 2 Department of Information Security Engineering, Soonchunhyang University, Asan 31538, Korea; vishal_sharma2012@hotmail.com; 3 Electronic Engineering Department, University of Seville, 41004 Seville, Spain; storal@us.es

**Keywords:** VANETs, genetic programming, broadcasting communications, dissimilarity metrics

## Abstract

This paper presents a novel dissimilarity metric based on local neighboring information and a genetic programming approach for efficient data dissemination in Vehicular Ad Hoc Networks (VANETs). The primary aim of the dissimilarity metric is to replace the Euclidean distance in probabilistic data dissemination schemes, which use the relative Euclidean distance among vehicles to determine the retransmission probability. The novel dissimilarity metric is obtained by applying a metaheuristic genetic programming approach, which provides a formula that maximizes the Pearson Correlation Coefficient between the novel dissimilarity metric and the Euclidean metric in several representative VANET scenarios. Findings show that the obtained dissimilarity metric correlates with the Euclidean distance up to 8.9% better than classical dissimilarity metrics. Moreover, the obtained dissimilarity metric is evaluated when used in well-known data dissemination schemes, such as p-persistence, polynomial and irresponsible algorithm. The obtained dissimilarity metric achieves significant improvements in terms of reachability in comparison with the classical dissimilarity metrics and the Euclidean metric-based schemes in the studied VANET urban scenarios.

## 1. Introduction

Data dissemination is a crucial operation in Vehicular Ad Hoc Networks (VANETs) [1]. The primary goal of a data dissemination algorithm is to efficiently spread out a given message from a source node to the rest of nodes in the network by exploiting multi-hop communications [2,3,4,5]. This type of operation is crucial for disseminating emergency messages in case of traffic accidents, which is one of the most important applications of data dissemination in VANETs [6,7]. Although many algorithms can be found in the literature [1], there is not a universal approach suitable for all possible cases. In general, data dissemination algorithms can be classified as deterministic and probabilistic algorithms, being probabilistic algorithms suitable in dynamic scenarios like VANETs, as given in prior studies [8,9,10,11]. Vehicular systems can be assisted through next generation of wireless networks (5G Infrastructure Public Private Partnership (5G PPP)) as well as onboard sensors for deciding the probabilistic as well as the deterministic flow of information across the network [12,13,14]. Moreover, onboard sensors help to localize the vehicles, which is required for the efficient applicability of data dissemination solutions [15,16]. 

Many topological parameters have been used in VANETs to heuristically calculate the suitable nodes’ retransmission probability such as the distance among nodes [17,18,19], local density estimators [20] like the number of neighbors, and the relative speed of nodes [21], among others [1]. Although it is not clear which one is best, it can be stated that the Euclidean distance is a good candidate for VANETs, since it is expected that in the near future the majority of cars will include a positioning sensor like Global Positioning System (GPS). Therefore, vehicles will be able to exploit their relative distance to disseminate information in a multi-hop fashion. Generally, the main benefit behind using the Euclidean distance to disseminate messages in a VANET is to give higher priority to those distant vehicles placed at the edge of the vehicles’ communication area. Consequently, the neighbors of a vehicle positioned at the edge of the wireless communication range are likely to be selected as forwarding nodes. Conversely, neighbor nodes located at a closer distance with respect to a source node will be silent to avoid redundant messages and network congestion. This mechanism is commonly known as silencing approach [22,23].

Most of the existing data dissemination algorithms for VANETs employ the ratio among the distance between two vehicles (sender and receiver) and the vehicle’s wireless communication range, which is considered to have the same value for all the vehicles in the VANET [18,19], to determine the potential forwarding nodes. However, such hypothesis of being aware of the actual value of the vehicle’s wireless communication range cannot be applicable in real VANETs due to a number of significant and uncontrollable factors. In general, vehicles in VANETs may have different wireless communication ranges depending on their onboard sensors [19]. Notice that it may even occur in the unlikely case that all vehicles use the same wireless transceiver because of production discrepancies. Furthermore, the vehicle’s wireless communication range in actual VANET scenarios may depend on uncontrollable and extremely changeable parameters, such as density of vehicles, interferences, and obstacles [24], among others. For these reasons, alternative approaches that select distant nodes using the vehicle’s communication range for such selection have been proposed [24,25,26]. On this line, dissimilarity metrics or distances, which employ the relation between the neighbors of two vehicles in order to calculate the similarity and/or dissimilarity between them [24,27], can be promising alternatives. The main rationale behind using dissimilarity metrics based on neighboring information is that two vehicles are similar if they share many neighbors, and therefore, two vehicles are likely to be similar if they are located at a closer distance. Conversely, the vehicles are dissimilar if they do not share neighbors. It has been demonstrated that classical dissimilarity metrics correlate moderately well with the Euclidean distance [24]. In general, the dissimilarity between two vehicles increases with respect to the relative Euclidean distance between them. Thus, dissimilarity metrics can be suitable estimators of the relative Euclidean distance among vehicles in VANETs.

There are several possible classical dissimilarity metric expressions that can be used to obtain an estimation of the relative distance among vehicles [24,27], such as Jaccard, Dice, and Sokal, among others. They use local density terms among two vehicles (sender and receiver) for example the number of neighbors that the two vehicles share and the number of neighbors that the two vehicles do not share, among others, to determine the similarity and/or dissimilarity between the two vehicles. A neighbor is shared by two vehicles if it is positioned within the wireless communication ranges of both vehicles. Furthermore, it is possible to obtain new dissimilarity metrics by determining new expressions that use the mentioned local density terms. However, finding the optimal one, that is, the one that best correlates with the Euclidean distance is a complex combinatorial optimization problem due to the number of possible relationships that can be used among the local density terms. In this paper, such combinational optimization problem is solved using evolutionary computation like a genetic programming (GP) approach.

### 1.1. Problem Statement

Data dissemination in VANETs should be performed efficiently in terms of reachability and network congestion. For that, a suitable data dissemination algorithm that selects appropriate forwarding vehicles to reduce the number of redundant packets should be employed. The ratio between the relative Euclidean distances between two vehicles with respect to the vehicle’s communication range is envisioned as the most suitable parameter to select forwarding vehicles in probabilistic data dissemination approaches. However, obtaining the actual vehicle’s communication range in real scenarios is unlikely to be effectively achieved due to the varying and dynamic behavior of the wireless medium. Therefore, it is necessary to find alternative metrics that are able to estimate the relative distance between vehicles and substitute the Euclidean distance in well-known data dissemination schemes like p-persistence approach [18].

### 1.2. Our Contribution

This paper proposes the use of genetic programming to obtain suitable dissimilarity metrics to be used in probabilistic data dissemination schemes as an appropriate alternative of the Euclidean distance. To the best of the authors’ knowledge, it is the first work on this line and it can pave the way of new research directions in the use of genetic programming to obtain suitable communication protocols in multi-hop network such as routing protocols and broadcasting approaches. Furthermore, this proof-of-concept has been validated in VANETs urban scenarios. The obtained dissimilarity metrics have been compared with classical dissimilarity metrics, achieving significant improvements in terms of reachability with respect to a number of well-known data dissemination algorithms for VANETs such as p-persistence, polynomial, and irresponsible algorithms.

## 2. Related Works

The related work section has been divided into two parts. First, we describe the main survey literature about data dissemination algorithms for VANETs. Second, we detail the main approaches proposed base on Euclidean distance and dissimilarity metrics.

### 2.1. Data Dissemination Algorithms in VANETs

Data dissemination algorithms based on broadcast procedure can be classified in several ways, such as single-hop versus multi-hop, deterministic versus probabilistic [8], and vehicle-to-vehicle versus vehicle-to-infrastructure communications [9]. Regarding the present work, the proposed data dissemination is based on multi-hop communications since vehicles will exchange information in a multi-hop fashion to calculate the dissimilarity metrics from the source vehicle until every vehicle has received a given message. The multi-hop procedure is preferred when the target application requires a high dissemination throughout the network, for instance, when safety messages should be spread out. Probabilistic approaches are suitable for robust communications since they give to each vehicle in the network the opportunity to participate in the data dissemination. They are also preferred in case of high-density scenarios. Furthermore, in this work only communications among vehicles are considered, therefore, we do not consider vehicle-to-infrastructure communications. In [9], the authors also classify the data dissemination algorithms with respect to the target VANET scenario layouts, such as highway and urban scenarios. The data disseminations algorithms present different features depending on the target layout. For instance, the speed of vehicles plays an important role in case of highways scenarios, while it is expected a moderately low speed of vehicles in case of urban scenarios. In this work, we focus on urban scenarios, where mobility of vehicles is low with respect the vehicles’ transmission range.

In ref. [10], the authors identify three models for data dissemination in VANETs, such as push, pull, and hybrid. The main difference between push and pull policies is that in pull techniques the messages are generated on the demand from the source vehicle, while in push techniques the messages are generated periodically. In this work, we focus on pull techniques, which require lower overhead. Another classification can be found in [11], where the techniques are categorized as forwarding based dissemination, broadcast based dissemination, push based dissemination, routing protocols based dissemination, and other dissemination mechanism. In forwarding algorithms, vehicles use techniques such as data aggregation and utility functions to determine the suitability of the retransmission of a message. Broadcast algorithms refer to the broadcast procedure used to disseminate the information, which is different with respect to the unicast mechanism used by routing based dissemination approaches. Regarding push based algorithms, they employ a dissemination policy considering the target application to determine the potential recipients of the messages. Regarding this work, the proposed approach is related to broadcast based dissemination using a utility function based on dissimilarity metrics.

Furthermore, there are available other seminar works on broadcasting for multi-hop networks that can be also applied to VANET scenarios. In ref. [11], the authors present a comprehensive survey on broadcast approaches for ad hoc networks. It describes broadcasting techniques that can be used in VANETs, such as counter-based approaches, area-based approaches, and neighbor knowledge approaches. In counter-based approaches, nodes counter the number of messages received as a local estimator of the local density of nodes. This metric is used for adjusting the nodes’ retransmission probability. In area based-approaches, nodes only retransmit incoming messages towards target areas. In neighbor-knowledge approaches, nodes determine the relationship among nodes to determine graph theory properties, such as connected dominant set (CDS) and self-pruning strategies.

Many variables have been used to determine the potential forwarding nodes in data dissemination algorithms in multi-hop network. However, it is not clear yet which parameter is the best one since there are not real experimentation-based comparisons among a wide range of algorithms in the literature. However, it is expected that the distance among nodes will be a relevant parameter to take into account since in the algorithms based on it, the number of retransmission has been reduced significantly in the network.

### 2.2. Probabilistic Data Dissemination Algorithms for VANETs Based on Distance

Many probabilistic data dissemination schemes that use the relative Euclidean distance among vehicles in VANETs can be found in the literature [18,19,23,28]. In ref. [18], the authors study the performance of p-persistence scheme for VANET scenarios, which determines lineally the retransmission probability with respect to the relative Euclidean distance between two vehicles and the vehicle’s wireless communication range, which is assumed to be equal for all the vehicles in the VANET. The p-persistence scheme employs the following expression:

(1) $$p=\frac{d_{ik}}{R}\;0<d_{ik}<1$$

where ***p*** is the retransmission probability of the receiver to retransmit an incoming message, ***R*** is the vehicles’ wireless communication range and ***d_ik_*** is the relative Euclidean distance between two vehicles ***i*** and ***k***. A simple evolution of p-persistence scheme is the polynomial scheme [22], which uses an exponent ***b*** to calculate the retransmission probability according to the following equation:

(2) $$p={\left(\frac{d_{ik}}{R}\right)}^{b}$$

Moreover, in [28] the authors propose irresponsible scheme, a probabilistic scheme based on the Cumulative Distribution Function (CDF) of the Euclidean distance between two vehicles. The main idea of irresponsible forwarding is that a vehicle should not retransmit an incoming message if it is probable that there is another node located farther away from the sender. The following expression is used by irresponsible scheme to determine the retransmission probability:

(3) $$p={\left(1-F_{x_{ij}}\left(R-d_{ik}\right)\right)}^{1/v}$$

$F_{x_{ij}}$ is the CDF of the Euclidean distance between two vehicles ***i*** and ***k***, and ***v*** is a tuning parameter to adjust the retransmission probability. The value of $F_{x_{ij}}$ depends on the spatial distribution of the vehicles in the VANET. In ref. [18], the authors demonstrate that exponential and lognormal distributions are likely to be found in VANET scenarios. In the case of the exponential distribution, the retransmission probability can be determined as follows:

(4) $$p=exp\left(-\frac{\rho_{s}\left(R-d_{ik}\right)}{v}\right)$$

where $\rho_{s}$ is the spatial density of vehicles in the VANET (vehicles/meter) and ***v*** the mentioned adjusting parameter. Other data dissemination schemes based on the irresponsible forwarding scheme can be found in [19,23]. In ref. [19], the authors modified the expression (4) to reflect that vehicles may have different wireless communication ranges. The resulting expression is:

(5) $$p=exp\left(-\frac{\rho_{s}\left(t-d_{ik}\right)}{v}\frac{R}{R_{i}}\right)$$

where ***R_i_*** is the wireless communication range of the ***i***th receiving vehicle and the ratio ***R***/***R_i_*** represents the differences in the vehicles’ wireless communication areas.

In [23], the authors improve the silencing mechanism proposed and analyzed in [22]. The idea is to use the relative Euclidean distance among vehicles to enhance the silence mechanism. In ref. [24], the authors demonstrate that dissimilarity metrics can also be suitable parameters to improve the performance of the silence mechanism.

All reviewed data dissemination scheme for VANETs based on the Euclidean present the same problem; all of them rely on the hypothesis that the wireless communication range of vehicles can be known. However, in real testbeds, it has been shown that the actual wireless communication range of a wireless node can be very different from the nominal range [29]. Extremely and external factors such as manufacturing mismatch between wireless transceivers, interferences among wireless technologies, noise, obstacles, density, and mobility may affect the actual wireless communication range. Consequently, the aforementioned Equations (1)–(5) will not reflect the actual relative distance between two vehicles. This problem can be solved by using dissimilarity metrics based on the local vehicles’ neighboring information.

The use of dissimilarity and/or similarity metrics [27] to improve probabilistic data dissemination in mesh networks has already been proposed in [24,25]. In ref. [25], the authors improve the discovery phase of the AODV routing protocol by using the Jaccard metric to determine the retransmission probability in order to reduce the congestion of the network. More recently in [24], the authors demonstrate experimentally that the use of dissimilarity metrics is suitable for improving the performance of probabilistic schemes like the p-persistence scheme based on the Euclidean distance. Furthermore, in [30] the authors evaluate probabilistic data dissemination algorithms based on dissimilarity metrics in VANET scenarios with Manhattan mobility model. The results in [30] demonstrate that dissimilarity metrics can solve the main problems raised by the use of the Euclidean distance to determine the retransmission probability in data dissemination schemes for VANET scenarios. In addition, in [30] the authors demonstrate that p-persistence scheme is the best one for VANET scenarios with intermediate density.

This work is a clear step forward with respect to the previous [24,25,30]. First, we propose new dissimilarity metrics obtained through genetic programming, which has not been applied to this research field so far. Second, we evaluate the obtained dissimilarity metrics in realistic standard VANET scenarios based on real city maps.

## 3. Dissimilarity Metrics Based on Neighboring Information

**Figure 1:**
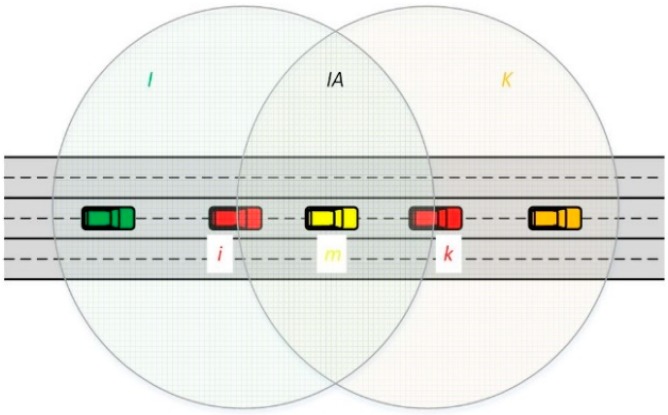
An illustration of a shared node between two vehicles. The vehicle in yellow is shared by both ***i*** and ***k*** vehicles.

The similarity and/or dissimilarity between two vehicles in a VANET can be calculated by employing local density metrics like the number of shared neighbor vehicles. A neighbor vehicle *m* is shared by two ***i*** and ***k*** vehicles (sender and receiver or vice-versa), if *m* is placed inside the wireless communication ranges of ***i*** and ***k*** (see Figure 1). It is worth indicating that the probability of a neighbor vehicle ***m*** of being within the overlapping area (IA in Figure 1) of both vehicles ***i*** and ***k*** depends on the Euclidean distance ***d*** between both vehicles and the density of vehicles in the VANET. In general, the size of IA decreases with the relative distance between two vehicles. Consequently, it is more probable to find more vehicles in the IA for a given VANET scenario. In addition, the dissimilarity between two vehicles can be defined as the contrary of the similarity. Notice that these definitions of similarity and dissimilarity between vehicles are also valid in case that the wireless communication range of vehicles is not an ideal circle as represented in Figure 1. Therefore, it is an important feature of the use of dissimilarity metrics, since they do not rely on wireless technology parameters or ideal circumstances. Consequently, they will adapt their performance to the real conditions in terms of connectivity among vehicles.

In general, the dissimilarity of two vehicles can be a suitable estimation of the relative Euclidean distance between two vehicles [24,25]. Most classical similarity/dissimilarity metrics are derived from the following general expression [27]:

(6) $$S_{ij}=\frac{a_{ik}}{a_{aik}+\lambda \left(a_{k}+a_{i}\right)}$$

where ***a_ik_*** accounts for the number of neighbor vehicles shared by the vehicles ***i*** and ***k***, the term ***a_i_*** determines the number of neighbor vehicles of the vehicle ***i*** that are not neighbors of the vehicle ***k***, and ***a_k_*** is the number of neighbor vehicles of the vehicle ***k*** that are not neighbors of the vehicle ***i***. With respect to Figure 1, ***a_ik_*** is the number of vehicles placed within the region *IA*, ***a_i_*** is the number of vehicles inside the *I* vehicle’ communication range (I in Figure 1), and ***a_k_*** is the number of vehicles within the region ***K***.

### 3.1. Definition of Classical Dissimilarity Metrics

Depending on the value of **λ** in (6) several classical similarity/dissimilarity metrics can be obtained. In addition, it is essential to notice that the relation between a similarity metric *S* and its equivalent dissimilarity metric DM is DM = 1 – *S* if *S* ∈ [0, 1].

The classical similarity and/or dissimilarity metrics can be formulated as follows:

Jaccard metric: This similarity metric ***Jac*** and distance ***Jacd*** between two vehicles ***i*** and ***k*** can be formulated as:

(7) $$Jac=\frac{a_{ik}}{a_{ik}+a_{i}+a_{k}}$$

(8) $$Jacd=1-J=\frac{a_{i}+a_{k}}{a_{ik}+a_{i}+a_{k}}$$

Dice metric: This similarity metric ***Dic*** and distance ***Dicd*** between two vehicles ***i*** and ***k*** can be expressed as:

(9) $$Dic=\frac{2a_{ik}}{2a_{ik}+a_{i}+a_{k}}$$

(10) $$Dicd=1-Dic=1-\frac{2a_{ik}}{2a_{ik}+a_{i}+a_{k}}=\frac{a_{i}+a_{k}}{2a_{ik}+a_{i}+a_{k}}$$

Kulczynski metric: This similarity metric ***Kul*** and distance ***Kuld*** between two vehicles *i* and *k* can be formulated as:

(11) $$Kul=0.5\left(\frac{a_{ik}}{a_{ik}+a_{i}}+\frac{a_{ik}}{a_{ik}+a_{k}}\right)$$

(12) $$Kuld=1-Kul=1-0.5\left(\frac{a_{ik}}{a_{ik}+a_{i}}+\frac{a_{ik}}{a_{ik}+a_{k}}\right)$$

Fowlkes-Mallows metric: This similarity metric ***Fow*** and distance ***Fowd*** between two vehicles ***i*** and ***k*** can be expressed as:

(13) $$Fow=\frac{a_{ik}}{\sqrt{\left(a_{ik}+a_{i}\right)\left(a_{ik}+a_{j}\right)}}$$

(14) $$Fowd=1-Fow=1-\frac{a_{ik}}{\sqrt{\left(a_{ik}+a_{i}\right)\left(a_{ik}+a_{k}\right)}}$$

Sokal-Sneath metric: This similarity ***Sok*** and dissimilarity ***Sokd*** can be determined as:

(15) $$Sok=\frac{a_{ik}}{a_{ik}+2\left(a_{i}+a_{k}\right)}$$

(16) $$Sokd=1-Sok=\frac{2\left(a_{i}+a_{k}\right)}{a_{ik}+2\left(a_{i}+a_{k}\right)}$$

### 3.2. New Dissimilarity Metrics

The aim of this paper is to find a more general expression for the dissimilarity metric not restricted to the general form given by (6) and best suited to the problem of replacing the Euclidean distance when determining their transmission probability of nodes in data dissemination schemes. A GP approach is used as a search engine for obtaining such better dissimilarity metric.

## 4. Genetic Programming

Genetic programming is a type of evolutionary algorithm inspired by biological evolution whose main objective is to find a computer program that performs a given task optimally. Since GP is based on the evolutionary methodology used by classical genetic algorithms (GAs) [25], it shares many properties with them such as genetic operators (selection, crossover, and mutation), initial population, fitness function, parents’ selection, elitism, and stop criterion. The main difference with respect to a GA is that in GP each individual represents a piece of code to be executed instead of the optimized variables of a given optimization problem.

Let us illustrate such difference with a simple example. If we consider the polynomial probabilistic data dissemination scheme [22], we can run a GA to obtain the best value of the exponent ***b*** (see Equation (2)) for a given scenario. However, the exponential relation between the Euclidean distance between two vehicles and the retransmission probability may not be the best one for the considered scenario. Then, we may run a GP algorithm to find the best relation between the Euclidean distance and retransmission probability (exponential, logarithmic, etc.). Therefore, when using a GP approach, we do not seek for the optimal values of certain variables or tuning parameters; we search for their optimal relation instead. In our case, the objective of the GP is to determine the optimal combination among the aforementioned terms ***a_i_***, ***a_k_***, and ***a_ik_***.

In a GP algorithm, a random initial population of potential solutions or individuals evolve through a number of generations, creating new individuals (offspring) based on the quality or fitness of the current individuals, and by using genetic operators, such as selection, crossover, and mutation. Algorithm 1 contains the pseudo code of the GP algorithm used in this letter.

**Algorithm 1.** Genetic programming.1: Objective function = PCC(M, D)2: Encode the solution into a tree (string)3: Generate the initial population4: Set crossover (pc) and mutation (pm) probabilities5: While (t < Max. of generations)6: Parents selection7: Crossover with pc8: Mutation with pm9: Evaluate offspring10:  Update t = t + 111: End While12: Decode the results and visualization

### 4.1. Representation of the Solutions

**Figure 2:**
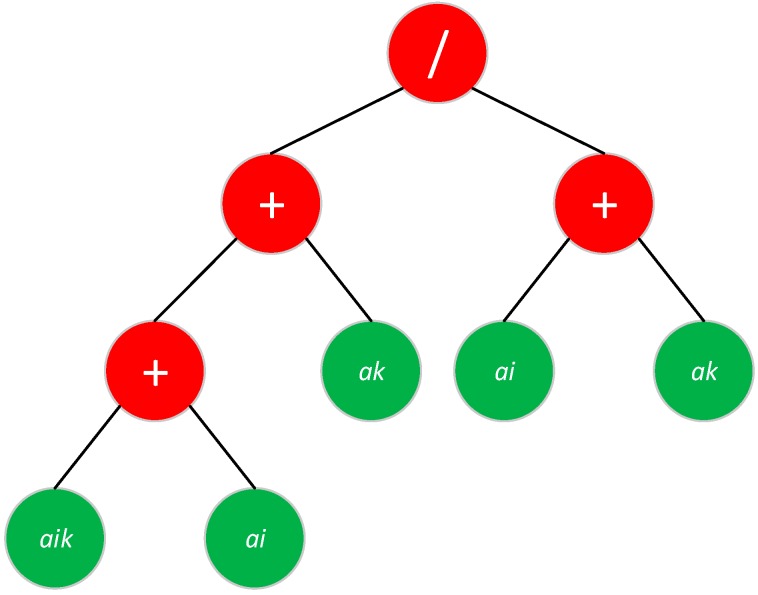
Example of solution tree for Jaccard dissimilarity metric.

Normally, the solution provided by a GP algorithm is represented as a tree, which depicts the steps and operations among the variables. Figure 2 illustrates the tree for the Jaccard dissimilarity metric (see Section 3).

The leaves of the three are the ***a*** terms described in Section 3 and the red nodes determine the relationships among ***a*** terms. In this paper, the primitive set of operations is composed of {summation, subtraction, multiplication, safe division, safe root square, safe logarithm}. Safe operators avoid the singularities of division by zero and root square and logarithm of a negative number. Furthermore, the depth of the tree and the number of operations is limited for computational reasons. Otherwise, the GP would converge to a solution impossible to implement (see Section 5 for more details).

In addition, another constraint is imposed to the individual generation. The values of the new dissimilarity metric between two vehicles in the VANET should be within the interval [0, 1]. This constraint can be formulated as:

(17) $$f_{j,k}\left(a_{ik},a_{i},a_{k}\right)\leq 1,\;\forall j,k\;s.t.\;d_{j,k}<R$$

Being ***d*** the Euclidean distance and ***R*** the vehicle’s communication range. Notice that this constraint stems from how the classical dissimilarity metrics are defined [27]. Nevertheless, it is suitable for the application of dissimilarity metrics in probabilistic data dissemination algorithms [30].

### 4.2. Fitness Function

The fitness function determines the quality of a potential solution. The Pearson Correlation Coefficient (PCC) is used to determine the correlation between the new dissimilarity metrics and the Euclidean distance. The PCC between two samples of continuous variables ***X*** and ***Y*** is calculated as:

(18) $$CC=\frac{cov\left(X,Y\right)}{\sigma x\sigma y}$$

where ***cov***(***X***,***Y***) is the covariance between ***X*** and ***Y***, and ***σ*** is the standard deviation. In this paper, the fitness function is obtained by averaging out the absolute value of ***PCC***(***M***,***D***) in several representative VANET scenarios obtained by varying the density of nodes (more details in Section 5). ***M*** represents the sample of dissimilarity values between each pair of neighbor nodes for a given new metric $m=f\left(a_{ik},a_{i},a_{k}\right)$, and ***D*** is the sample of the Euclidean distances among each pair of neighbor nodes. Two vehicles are neighbors if they are within the wireless communication range of each other, so ***d*** < ***R***. Therefore, the fitness function of the GP implementation is:

(19) $$F=\frac{1}{n}\sum_{i=1}^{n}abs{\left(PCC\left(M,D\right)\right)}_{i}$$

where ***n*** is the number of VANET scenarios. Thus, the optimization problem is defined as:

(20) Maximize Fs.t.(17)

Notice that the fact of considering the absolute values of PCC makes possible to obtain both similarity and dissimilarity metrics.

### 4.3. Genetic Operators

They are responsible for generating new individuals (offspring) from the current population. There are two main genetic operators in GP such as crossover and mutation. By applying crossover, the genetic information of two individual is exchanged. The objective of crossover is to explore new solutions in the search space. On the other hand, mutation consists of slightly varying the genetic information of an individual. Consequently, the vicinity of the current individual in the search space is explored. Both genetic operators are applied according to a given probability. As a rule, the mutation probability is much lower than the crossover probability. Both probabilities are design parameters and must be carefully selected.

Selection is based on tournament mechanism, which has been demonstrated to be suitable for evolutionary computation. The crossover used is the one-point scheme. Therefore, the trees of two selected individuals are swapped using one point as a reference. Regarding mutation, the uniform scheme is used. In the uniform scheme, a random subtree of a solution is replaced by a new random one with the same depth. Notice that both crossover and mutation are probabilistic operations (*p_c_* and *p_m_* in Algorithm 1) and along with the selection mechanism, they determine the exploration and exploitation power of the optimization GP algorithm. Since there is no consensus in the literature regarding the optimal values of *p_c_* and *p_m_*, several values have been tested in Section 5.

### 4.4. Stopping Criterion and Time Complexity

It determines when the GP stops. Normally the number of generations is the parameter used to stop the evolution of a GP [31]. The time complexity of the algorithm is determined by two parameters, such as number of generations *G* and the number of individuals ***I***. The number of simulations required to obtain a suitable solution is ***G*** × ***I***. Appropriated values should be selected in order to achieve suitable solutions in a reasonable time. Further details about the selected values are given in the next section.

## 5. Simulation Results

**Figure 3:**
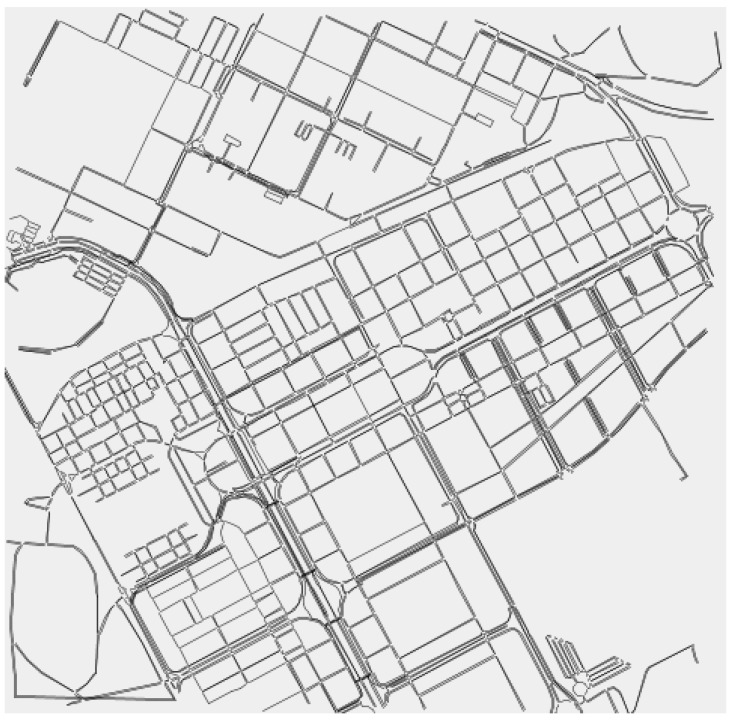
The VANET scenario considered for the simulations.

Numerical results have been obtained with a simulator developed in Python [32]. The GP algorithm has been implemented in DEAP [33], a Python module for evolutionary algorithms. Since the number of simulations required by the GP-based approach is high, we have developed a pure and lightweight simulator that integrates well with the DEAP module. This is the main reason for using the simulation available in [32], rather than using other network simulators for VANETs such as NS-2 and NS-3, which requires considerably more computation resources. Regarding the GP parameters, the population size is 100 individuals, and up to three depths of the tree have been evaluated, such as 4, 5, and 6. The ***p_c_*** used is within the interval [0.6, 0.8] with a step of 0.1. The *p_m_* tested is within the interval [0.05, 0.2] with a step of 0.5. Therefore, 12 different parameter settings of the GP have been evaluated. The limit of operations among the *a* terms is 100.Both the depth of the trees and the number of operations reduces the algorithmic complexity of the proposed approach. Higher values of both terms will lead to solutions very difficult to implement in real-life scenarios. The number of generations is 100 and each setting is evaluated with 10 different seeds of random numbers. Regarding the VANET scenarios, they are based on real maps using C4R tool [34]. The density of vehicles varies within the interval [100, 200] in steps of 10. The VANET scenario is based on a real map of the city of Seville in Spain, which is shown in Figure 3. The size of the scenario is 2 km × 2 km. The vehicle’s communication range ***R*** is 250 m and the unit disk model is used. To measure the correlation, we consider that the vehicles are static during the simulation. Notice that this assumption will not affect the correlation results between the Euclidean distance and the obtained dissimilarity metric in real mobile scenarios.

### 5.1. Correlation Results

This section contains the correlation results among the dissimilarity metrics obtained using the GP and the Euclidean distance. Table 1 contains the correlation results achieved by the best dissimilarity metrics obtained by the GP for the three depths considered. The GP parameter settings that lead to the best results are shown in Table 1. Traditional dissimilarity metrics have been included for the sake of comparison.

**Figure 4:**
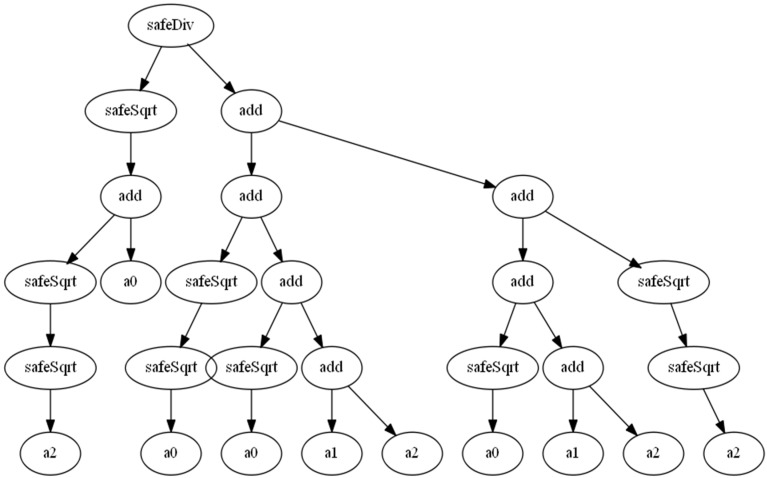
Best solution obtained by the GP approach, ***a*_0_** = ***a_ik_***, ***a*_1_** = ***a_i_***, and ***a*_2_** = ***a_k_***.

Figure 4 shows the best solution obtained. According to Table 1, it correlates with the Euclidean distance better than classical dissimilarity metrics in 8.9%. More details about other solutions obtained can be accessed at [32]. In regard to the expression for the best achieved metric is:

(21) $$S_{new}=\frac{\sqrt[{4}]{a_{k}}+a_{ik}}{{\left[a_{ik}\sqrt[{4}]{a_{ik}}+2\left(a_{i}+a_{k}\right)+\sqrt[{4}]{a_{k}}\right]}^{2}}$$

**Figure 5:**
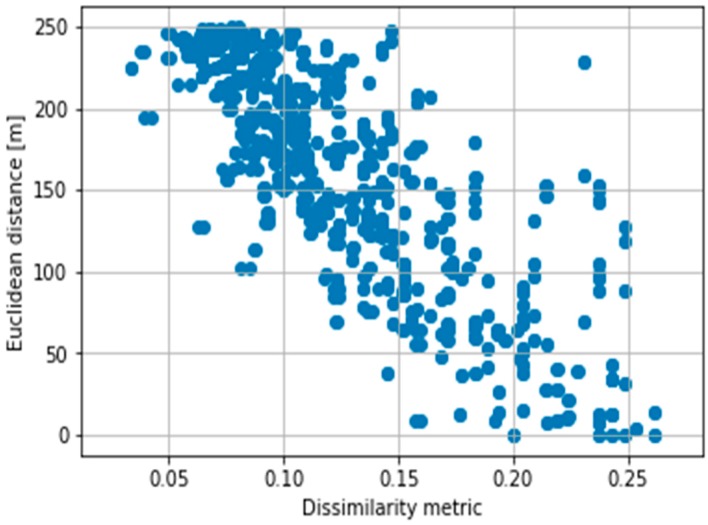
Euclidean distance versus dissimilarity metric for 120 vehicles.

It is interesting the appearance of the term $2\left(a_{i}+a_{k}\right)$ in the divisor. It also appears in Sokal-Sneath expression (see Section 3), which achieves the best results among the classical dissimilarity metrics (see Table 1). Therefore, it plays an important role in the correlation with the Euclidean distance. Figure 5 shows the Euclidean distance versus the metric obtained for 120 vehicles. Figure 5 shows that the obtained metric is a similarity metric since it decreases with an increase in the Euclidean distance. Therefore, $DM_{new}=1-S_{new}$. Thus, $DM_{new}$ will be used to replace the Euclidean distance in the next section.

### 5.2. Data Dissemination Results

Now, the goodness of the obtained dissimilarity metric is evaluated under different probabilistic data dissemination schemes such as p-persistence algorithm, polynomial and irresponsible algorithm. The normalized Reachability (Re) and the number of redundant messages are used as performance metrics. The Re metric measures the percentage of vehicles that receive the broadcast message generated by the source nodes [24,30]. The normalization of both metrics is done by dividing by the results of flooding scheme, which is used as an upper bound. Re should be as high as possible since it is always a major requirement of an efficient data dissemination scheme. Regarding the number of redundant messages, it is desirable to achieve a low redundancy since it is related to the congestion of the network. In previous works [24,30], the p-persistence based on dissimilarity metric algorithm has been demonstrated to be the most appropriate for VANET scenarios with medium density levels such as the ones used in this letter. Therefore, p-persistence algorithm [18] is used as a baseline data dissemination algorithm to test the obtained metric. Algorithm 2 contains the pseudocode of the p-persistence algorithm based on the dissimilarity metric. *DMik* represents the dissimilarity metric between vehicles *i* and *k*, which is calculated using (6).

**Algorithm 2.** P-persistence algorithm based on dissimilarity metric.1: Whenever a message *g* is received1: If *g* is new:3: Retrieve neighboring list from *g*4: Calculate ***a_ik_***, ***a_i_***, ***a_k_***5: Calculate *p* as $p=DM_{ik}$6: If *p* ≥ Rand [0,1]7:  Include neighboring list in *g*8:  Rebroadcast *g*9: Else:10:   Eliminate *g*11:  End if12: Else:13:  Eliminate *g*12: End if

**Figure 6:**
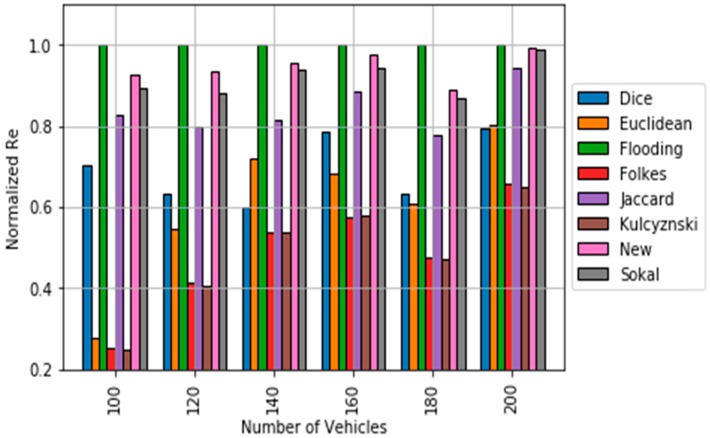
Comparison of Reachability results for the p-persistence scheme based on the different dissimilarity metric.

**Figure 7:**
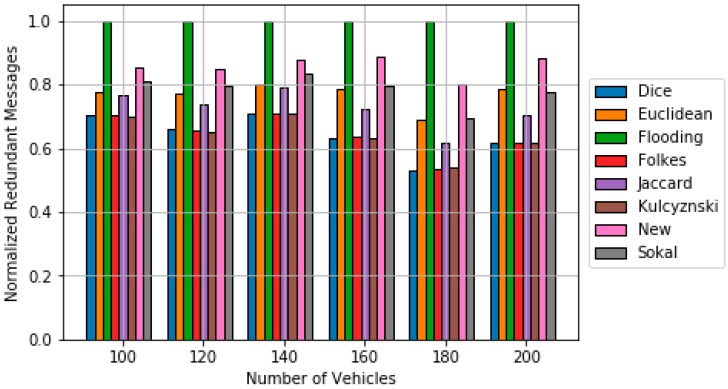
Comparison of redundancy results for the p-persistence scheme based on the different dissimilarity metric.

Figure 6 and Figure 7 show the obtained simulation results. The results are obtained by averaging out 20 different trials. Up to 30 random source nodes are selected in each trial [28]. It can be observed that the p-persistence algorithm based on the obtained dissimilarity metric achieves better results in terms of ***Re*** than in the cases that used classical dissimilarity metrics and the Euclidean distance. The percentage of improvement with respect to the best classical dissimilarity metric (Sokal-Sneath) is within the interval [0.7, 6]. In addition, the obtained new metric clearly outperforms the results of the Euclidean based p-persistence. Regarding redundancy, the proposed approach outperforms flooding and it is closed to Sokal-Sneth metric.

**Figure 8:**
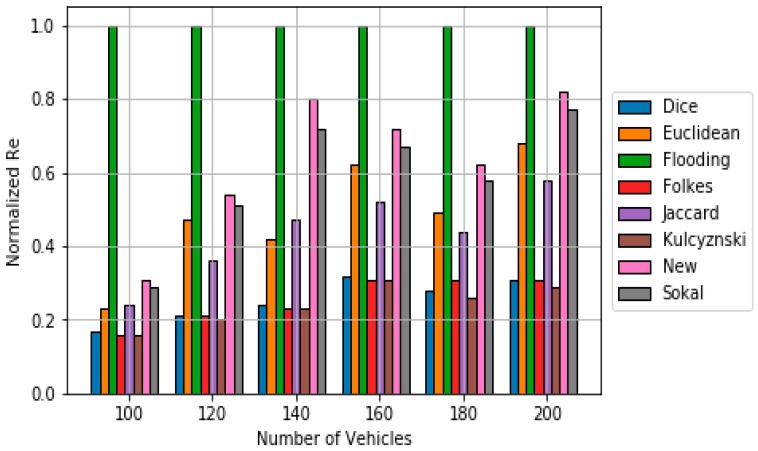
Comparison of Reachability results for the polynomial scheme based on the different dissimilarity metric.

**Figure 9:**
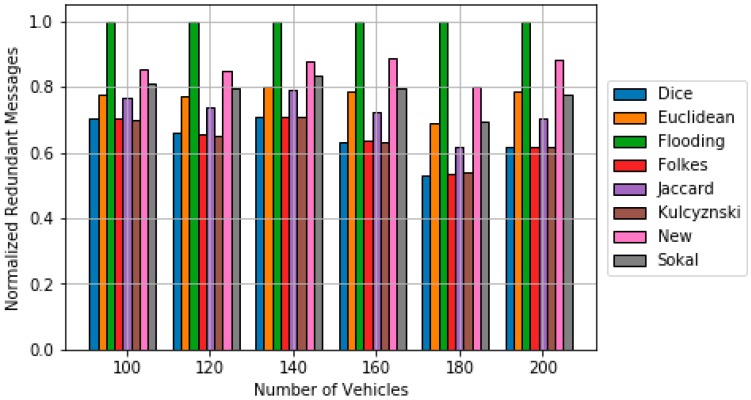
Comparison of redundancy results for the polynomial scheme based on the different dissimilarity.

Regarding polynomial scheme, Algorithm 3 contains the pseudocode used in this work. Again, *DMik* represents the dissimilarity metric between vehicles *i* and *k*, which is calculated using (6). The value of ***b*** is 2 since higher values of ***b*** have been already tested, showing low performances [30]. Figure 8 shows the obtained simulation results in terms of ***Re***. It can be observed that the majority of classical dissimilarity metrics do not work well when used in polynomial scheme. However, the new dissimilarity metric suits quite well when applied to polynomial scheme. However, it requires a higher number of messages to obtain a suitable Re (see Figure 9).

**Algorithm 3.** Polynomial algorithm based on dissimilarity metric.1: Whenever a message *g* is received1: If *g* is new:3: Retrieve neighboring list from *g*4: Calculate ***a_ik_***, ***a_i_***, ***a_k_***5: Calculate *p* as $p={\left(DM_{ik}\right)}^{b}$6: If *p* ≥ Rand [0,1]7:  Include neighboring list in *g*8:  Rebroadcast *g*9: Else:10:   Eliminate *g*11:  End if12: Else:13:  Eliminate *g*12: End if

**Figure 10:**
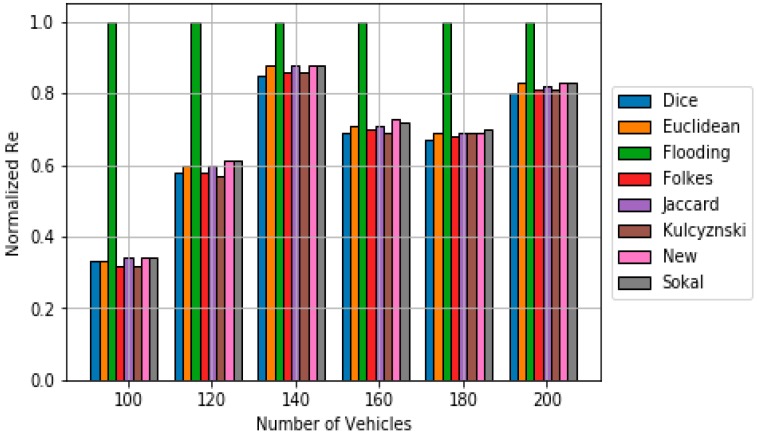
Comparison of Reachability results for the irresponsible scheme based on the different dissimilarity metric.

**Figure 11:**
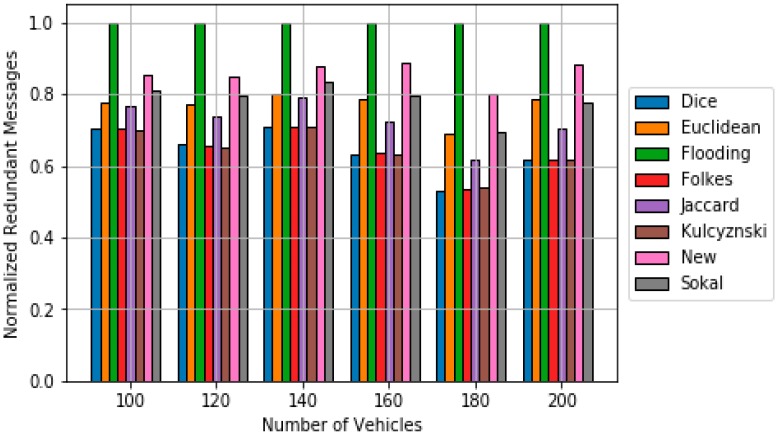
Comparison of redundancy results for the irresponsible scheme based on the different dissimilarity metric.

With regard to the irresponsible scheme, Algorithm 4 shows the actual implementation of the scheme based on dissimilarity metric. The scheme has been adapted since in the original expression (4), the retransmission probability depends on a global parameter like the density of vehicles $\rho_{s}$. Such global parameter cannot be obtained in a distributed way; therefore, the node’s number of neighbors has been used as a local estimator of the density. Regarding the shaping parameter ***v***, it has been fixed to 20, which has already been demonstrated to provide good results in terms of reachability [30]. Notice that the most optimal configuration of polynomial and irresponsible schemes is out of the scope of the paper. Figure 10 includes the obtained simulation results for irresponsible scheme in terms of ***Re***, which again validate the obtained dissimilarity metric. Figure 11 contains the obtained results in terms of number of redundant messages. The proposed approach outperforms flooding, but it requires a higher number of messages compared with classical dissimilarity metrics.

**Algorithm 4.** Irresponsible algorithm based on dissimilarity metric.1: Whenever a message *g* is received2: If *g* is new:3: Retrieve neighboring list from *g*4: Calculate ***a_ik_***, ***a_i_***, ***a_k_***5: Calculate *p* as $p=\exp\left(-\frac{n_{b}\left(1-(DM_{ik}\right)}{v}\right)$6: If *p* ≥ Rand [0,1]7:  Include neighboring list in *g*8:  Rebroadcast *g*9: Else:10:   Eliminate *g*11:  End if12: Else:13:  Eliminate *g*12: End if

### 5.3. Future Work

As possible future works, the authors plan the following research directions:

- Combine the proposed approach with online approaches based on learning policies like [35]. The idea is to reduce the number of messages exchanged among nodes by updating the hyper-parameters of learning models.
- Extend this work by evaluating the proposed approach in highways scenarios in order to assess how the high speed of vehicles affects the dissimilarity metric calculation while using modernized sensor setups [36,37].
- Evaluate the proposed approach under different wireless and sensor technologies for VANETs, such as IEEE 802.11p, IEEE 802.11ax, and IEEE 802.15.4, among others.
- Evaluate the proposed approach in other multi-hop networks, such as Mobile Ad Hoc Networks (MANETs), Delay Tolerant Networks (DTNs), and Flying Ad Hoc Networks (FANETs) [38,39].
- Since in majority of cases the proposed approach outperforms the other algorithms in terms of ***Re***, but with an increase of redundancy, we plan to extend the work by considering a multi-objective genetic programming approach [40]. Therefore, both reachability and redundancy can be balanced.

## 6. Conclusions

A new dissimilarity metric based on local neighboring information to be used in probabilistic data dissemination algorithms for VANET urban scenarios has been obtained thanks to a genetic programming approach. The suitable relationship among local density metrics, which is a complex combinatorial optimization problem, has been obtained by maximizing the correlation among the dissimilarity metric and the Euclidean distance for several representative and real-map based VANET scenarios. The simulation results show that the obtained novel dissimilarity metric achieves better correlation results than the classical dissimilarity metrics with respect to the Euclidean distance. In addition, it improves the performance in terms of reachability with respect to several well-known probabilistic data dissemination schemes for VANETs, such as p-persistence, polynomial, and irresponsible algorithms with respect to the original algorithms, which employ the relative Euclidean distance among vehicles. To the best of the authors’ knowledge, this is the first work that applies a GP approach to obtain metrics for data dissemination in multi-hop networks. Therefore, it may enable new research directions based on this line.

## Figures and Tables

**Table 1:** Correlation results.

Metric	Correlation
Jaccard	0.661357
Dice	0.622318
Kulczynski	0.616629
Fowlkes-Mallows	0.620337
Sokal-Sneath	0.620337
GP Metric (Depth = 4, *p_c_* = 0.7, *p_m_* = 0.1)	0.738470
GP Metric (Depth = 5, *p_c_* = 0.8, *p_m_* = 0.15)	0.741575
GP Metric (Depth = 6, *p_c_* = 0.8, *p_m_* = 0.2)	0.740777
